# Contrasting assembly processes in a bacterial metacommunity along a desiccation gradient

**DOI:** 10.3389/fmicb.2014.00668

**Published:** 2014-12-03

**Authors:** Angel Valverde, Thulani P. Makhalanyane, Don A. Cowan

**Affiliations:** Department of Genetics, Centre for Microbial Ecology and Genomics, Genomics Research Institute, University of PretoriaPretoria, South Africa

**Keywords:** bacteria, stochastic, deterministic, drought, community assembly

## Abstract

Understanding the relative influence of deterministic and stochastic processes in driving community assembly is a major goal in microbial ecology. Here, we have investigated the influence of these processes on bacterial community assembly in the lateral sediments of a salt pan along a desiccation gradient over a three-year period. We show that the role of deterministic processes increases in communities distant from the water line (shaped by drought), probably as a result of the interplay between abiotic and biotic factors. By contrast, the influence of stochastic processes on bacterial community assembly was higher in the sediments closest to the water line, more likely due to lower levels of abiotic stress. Our results demonstrate that both deterministic and stochastic processes influence bacterial community assembly in salt pan sediments, and that their relative influence varies along a desiccation gradient.

## Introduction

Unraveling the mechanisms that govern the assembly of microbial communities is an important goal in ecological research. There are two major contrasting conceptual frameworks explaining community assembly processes. The first, exemplified by the long-standing hypothesis “everything is everywhere, but the environment selects” (Baas-Becking, 1934), postulates that microbial communities are shaped by deterministic (biotic and abiotic) factors; whereas the second, conceptualized in the neutral theory (Hubbell, 2001), states that community assembly is simply the result of the stochastic processes of random births, deaths, migration and speciation. While different studies have suggested a dominant role of determinist processes in the assembly of microbial communities (Lozupone and Knight, 2007; Tamames et al., 2010); today, it is widely recognized that these two mechanisms are not mutually exclusive, but interact in the assembly of microbial communities (reviewed in Lindstrom and Langenheder, 2012; Hanson et al., 2012). Vellend's conceptual synthesis of community ecology (Vellend, 2010) illustrates this view: “species are added to communities via speciation and dispersal, and the relative abundances of these species are then shaped by drift and selection, as well as ongoing dispersal, to drive community dynamics.”

Nevertheless, in spite of these advances, the relative contribution of deterministic and stochastic processes in the assembly of microbial communities is still debated. For example, it has been suggested that microbial communities in more benign environments (e.g., those with higher productivity) are more likely to be shaped by neutral processes (Chase, 2010), whereas environmental selection is more plausible in communities thriving in high stress habitats (Wang et al., 2013). Clearly, more research is needed to better understand the balance between stochastic and deterministic processes and the effect of these processes on the abundances and composition of microbial communities.

Natural communities are made of species with different degrees of ecological specialization (i.e., habitat specialist and generalist species; e.g., Devictor et al., 2010). Environmental gradients might promote habitat diversification, which is likely to cause an increase in habitat specialists (that is, taxa with narrow environmental tolerance). In general, habitat specialists seem to be mostly influenced by deterministic factors (e.g., Pandit et al., 2009), while habitat generalists appear to respond mainly to stochastic factors (but see Székely and Langenheder, 2014).

Here, we have examined the bacterial community structure in a coastal salt pan (Yzerfontein, Western Cape, South Africa). The site is a brackish-saline pan rich in gypsum and characterized by seasonal drought and periodic strong offshore and onshore winds patterns, which make this habitat a good model to study community assembly. Aeolian dispersion may buffer the effect of environmental selection by continued homogenization of the communities involved (Mouquet and Loreau, 2003), while droughts are ecologically important disturbances that increase the effect of environmental selection in community assembly (Chase, 2007).

Using T-RFLP analysis of bacterial 16S rRNA genes, the concept of “indicator species” (Dufrene and Legendre, 1997) and β-diversity (turnover in taxa composition in space and time) patterns we aimed to study seasonal changes, if any, in bacterial community assembly processes. We sampled the bacterial community and analyzed the chemistry of sediments in the lateral zones of the pan, along a desiccation transect from the water line to the pan margins. We hypothesize that deterministic processes (environmental filtering through droughts) will be more important for bacterial community assembly in water-distant sites, whereas stochastic assembly processes (random dispersal, ecological drift) will prevail in bacterial populations in the vicinity of the water source. In addition, we expect that deterministically assembled communities should contain a larger proportion of habitat specialists.

## Materials and methods

### Site and climate description

Yzerfontein salt pan (Supplementary Figure S1) is a 116 ha coastal pan situated northeast of the town of Yzerfontein, about 80 km north of Cape Town (South Africa). It consists of a large playa of mudflats in a shallow depression (no more than 5 m above sea level), with a low, narrow ridge of calcareous sand dune preventing any water outflow. The region experiences cool wet winters (April to September) and hot dry summers (October to March) when arid conditions prevail and when evaporation significantly exceeds the summer annual rainfall of about 41 mm/yr (Supplementary Table 1).

Sediments were sampled on five different occasions between Nov-2011 and Feb-2014 (3 summers and 2 winters, Supplementary Table 1). 6 cm deep samples (from 2 to 8 cm depth) were collected using sterile 50-ml tubes. Typically, this layer contains gypsum, calcite, halite and quartz (Smith and Compton, 2004). Six different transects, covering the southern section of the pan, were sampled. For each transect, samples were collected at 1, 10, and 20 m from the water line to the pan margin (from wet to dry sites). At each site five subsamples were combined, homogenized in the field, and stored in sterile Whirl-Pack sample bags (Nasco, WI, USA). Bags were kept at 4°C, transported to the laboratory and processed within 2 days of sampling. The complete sample set contained 90 samples (6 transects × 3 sites × 5 time-points).

### Sediment chemistry

Samples were analyzed for total carbon, total nitrogen, major elements, pH, and percentage of sediment moisture. Light element analysis (%N, %C) was determined using a LECO Truspec elemental determinator. Major element analysis (Al_2_O_3_, CaO, Cr_2_O_3_, Fe_2_O_3_, K_2_O, MgO, MnO, Na_2_O, P_2_O_5_, SiO_2_, and TiO_2_) was determined using X-ray fluorescence spectrometry (Philips PW1404 XRF). pH was analyzed in a 1:1 water dilution. Water content was determined by weighing 10 g of sediments before and after oven drying at 60°C for 48 h. Determinations were performed at the Stellenbosch Central Analytical Facilities (Stellenbosch University, SA) using standardized procedures.

### DNA extractions and T-RFLP analysis

DNA was extracted from 0.5 g of sample using the MoBio PowerSoil DNA isolation kit (Mo BIO, Carlsbad, CA, USA). Bacterial 16S rRNA gene amplification was performed using primer pair 341F (Ishii and Fukui, 2001) and 908R (Lane et al., 1985), labeled with 6-FAM, as previously described (Valverde et al., 2012). PCR products were combined from three amplification reactions per sample, verified by agarose gel electrophoresis and purified with NucleoSpin Extract II (BD Biosciences Clontech, Japan). Approximately 200 ng of purified products were digested in separate reactions using *TaqI* and *HaeIII* restriction enzyme (Fermentas). After purification as above, samples were subjected to capillary electrophoresis using the Applied Biosystems DNA Sequencer 3130 (Applied Biosystems, Foster City, California, USA). Terminal restriction fragments (T-RFs) data generated by Peak Scanner software v1.0 (Applied Biosystems) were filtered and binned by the method developed by Abdo et al. (2006).

### Data analysis

Bacterial richness and evenness (Pielou's index), and abiotic raw data were compared using a Kruskal-Wallis test (Hollander and Douglas, 1973). Bacterial community structure was explored using qualitative (i.e., Jaccard) and quantitative [i.e., Bray-Curtis, Hellinger transformed (Legendre and Gallagher, 2001)] dissimilarity matrices. The underlying mechanisms of community assembly were explored using a modified Raup-Crick dissimilarity metric (Raup and Crick, 1979), which is robust to variations in local species richness (Chase et al., 2011). Raup-Crick metric expresses the compositional dissimilarity, using presence/absence data, between the observed communities relative to those generated under the null model, by estimating the probability that any two null communities drawn randomly from the “regional” species pool have the same number or more species in common than the observed communities. If Raup-Crick dissimilarity values (β_RC_) are not significantly different from 0 this indicates community assembly is stochastic. β_RC_ values approaching −1 indicate that communities are deterministically assembled and more similar than expected by chance due to strong habitat filtering, whereas β_RC_ values close to +1 indicate that deterministic factors (e.g., interespecies competition) favor dissimilar communities, or that dispersal between sites is very low (Chase et al., 2011). The null expectation was generated using 9999 randomizations using the “raupcrick” function in the vegan (Oksanen et al., 2013) package for R (R Development Core Team, 2013). We calculated the null deviation as the difference between the mean β_RC_ and zero, which indicates stochastic community assembly. We tested whether null deviation values were different from zero using a two-tailed Wilcoxon signed-rank test.

A permutational analysis of variance (PERMANOVA; Anderson, 2001) was used to test for differences in composition between habitats, whereas permutation dispersion (Anderson et al., 2006) was used to test for differences in their within-habitat dissimilarity, both using the vegan package. Indicator value (IndVal) indexes (Dufrene and Legendre, 1997) were calculated using function indval in the labdsv package (Roberts, 2013) for R. The temporal development of bacterial communities was analyzed using non-metric multidimensional scaling (nMDS) based on Bray-Curtis (Hellinger-transformed) dissimilarities. Mantel tests for correlations between environmental factors and bacterial communities were calculated using R (9999 permutations).

## Results and discussion

We investigated the bacterial communities, by means of T-RFLP analysis, in the margins of a coastal salt pan over 2 years in order to understand the relative influence of deterministic and stochastic processes in driving community assembly. T-RFLP analysis typically resolves taxa to near the species-level and detects OTUs with relative abundance >0.1% (e.g., Fierer and Jackson, 2006; Pedros-Alio, 2012). Therefore, an important limitation of our study is that, in contrast to results obtained through application of technologies such as clone libraries or next-generation sequencing (NGS), low abundant taxa are not detected by fingerprinting techniques (Bent and Forney, 2008). However, although NGS has a higher level of resolution, both fingerprinting and NGS analyses have often been found to generate comparable patterns of community composition (e.g., Besemer et al., 2012; Gobet et al., 2014, indicating that a fingerprinting method targeting the most abundant OTUs may lead to reliable β-diversity patterns.

**Figure 1:**
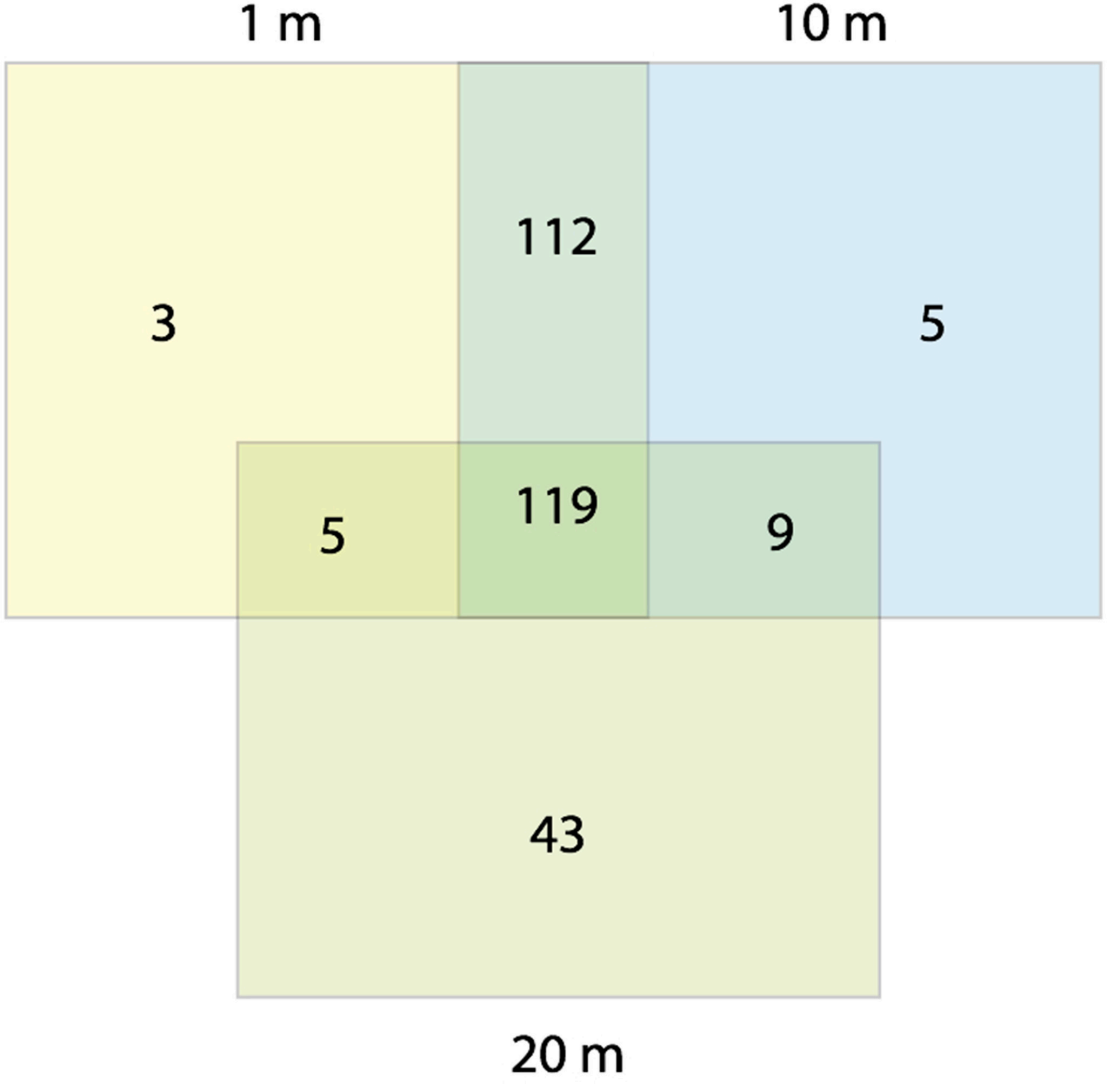
**Venn diagram showing T-RFLP derived OTUs per sampling site (1, 10, and 20 m from the water line)**.

Water content in the sediments decreased with increasing distance from the water line, although the differences were statistically significant only for the most distant samples during summer (Table 1). We therefore use the terms “dry” and “wet” to refer to communities distant 20 m, and 1–10 m from the water line, respectively. No statistically significant differences in chemistry between dry and wet samples were found. A total of 296 OTUs (operational taxonomic units) were observed across all samples (γ-diversity) (Figure 1), ranging from 20 to 60 OTUs for the individual samples [38 (mean) ± 8 (SD)]. OTU richness (α-diversity) and Pielou's evenness (0.40 ± 0.10) remained relatively constant in all three sediment habitats over time (Kruskal-Wallis test, *P* > 0.05). Wet sediments contained 245 (1-m distant samples) and 239 (10-m distant samples) OTUs, while dry sediments contained 176 OTUs, 28% lower γ-diversity than was observed in 1-m distant samples. Reductions in γ-diversity have previously been observed after environmental disturbances; for example, in ponds experiencing drought (Chase, 2007) and in soils after a fire event (Ferrenberg et al., 2013). Three OTUs were unique to the samples closest to the shoreline, while 5 and 43 were unique to the 10 and 20-m distant samples, respectively. When OTUs were analyzed by season, 11 were winter-specific, 24 summer-specific and 261 were shared. These numbers should be interpreted with caution, as fingerprinting techniques are known to underestimate community diversity (Bent and Forney, 2008). However, the main aim of this study was not to assess the true diversity in the samples, but rather to monitor β-diversity patterns.

**Table 1:** **Values of chemical parameters of the sediments sampled**.

**Distance to water**	**pH**	**WC (%)**	**Al_**2**_O_**3**_ (%)**	**CaO (%)**	**Fe_**2**_O_**3**_ (%)**	**K_**2**_O (%)**	**MgO (%)**	**MnO (%)**	**Na_**2**_O (%)**	**P_**2**_O_**5**_ (%)**	**SiO_**2**_ (%)**	**TiO_**2**_ (%)**	**C (%)**	**N (%)**
1 m	7.5	30^a^	1.31	22.69	0.59	0.28	2.00	0.01	5.34	0.09	23.59	0.08	2.57	0.04
10 m	7.6	25^a^	1.18	23.27	0.50	0.25	1.48	0.01	4.07	0.07	25.10	0.08	2.03	0.02
20 m	7.6	10^b^	1.18	24.87	0.47	0.26	1.50	0.00	4.67	0.07	18.25	0.07	1.71	0.03

WC, Water content. Different superscript letters indicate significant differences between samples (P < 0.05).

Values are presented as means.

**Figure 2:**
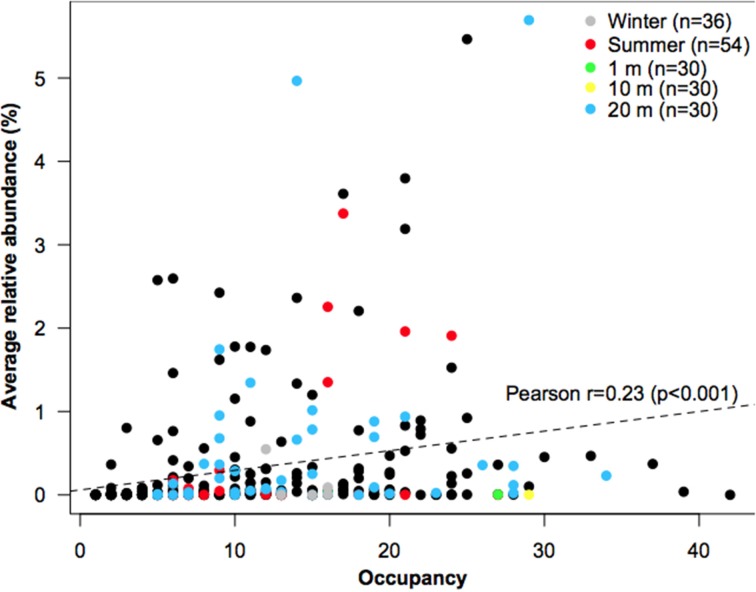
**Average relative abundance (y-axis) and occupancy (x-axis) of all OTUs**. Colored dots represent top indicator OTUs for each environment, with indicator values >0.3 and *P* < 0.01. The number of samples for each environment is denoted in the legend.

Based on the frequency of OTUs generated using T-RFLP, four out of ten bacterial taxa were found in both dry and wet samples, which suggest that a great number may thrive under both conditions (widespread taxa, so-called habitat generalists). There was also a positive interspecific abundance-occupancy relationship (Figure 2), one of the most robust patterns observed for both micro- (Nemergut et al., 2011) and macro-organisms (Verberk et al., 2010). Nevertheless, based in the concept of indicator species (Dufrene and Legendre, 1997), it was possible to determine that there exist dominant taxa in each environment (habitat specialists) (Figure 2); although larger numbers of habitat specialists were detected in 20-m distant samples. Therefore, it appears that dry conditions have selected more strongly for specific OTUs, which agrees with the concept that habitat specialists respond more to environmental filtering (Pandit et al., 2009; Logares et al., 2013). Notably, a large number of habitat specialists OTUs showed comparatively low relative abundances, which seems to contradict the definition of habitat specialist (i.e., locally abundant taxa; Barberan et al., 2011). However, low abundance can result from specialization, as this trait may imply a more restrictive use of available resources (Vazquez and Aizen, 2003). Taken together, these results indicate that a portion of the metacommunity was shaped by environmental filtering. Nevertheless, as several taxa were ubiquitous across both sample sites and seasons, it seems that stochastic conditions (e.g., wind, rainfall events) also play a role in bacterial community assembly, especially for bacterial communities in “wet” habitats. A plausible reason for this may be the influx of bacteria from the pan due to a better hydrologic connectivity between the pan and the wet sediments or to water run-off from the upper (dry) sediments. Once interstitial pore spaces become filled with water after a precipitation event, patches may become connected and surface flow downstream will occur, as has been shown in artic freshwaters inoculated from soils (Crump et al., 2012). Alternatively, these habitat generalist taxa may be responding to other environmental factors.

**Figure 3:**
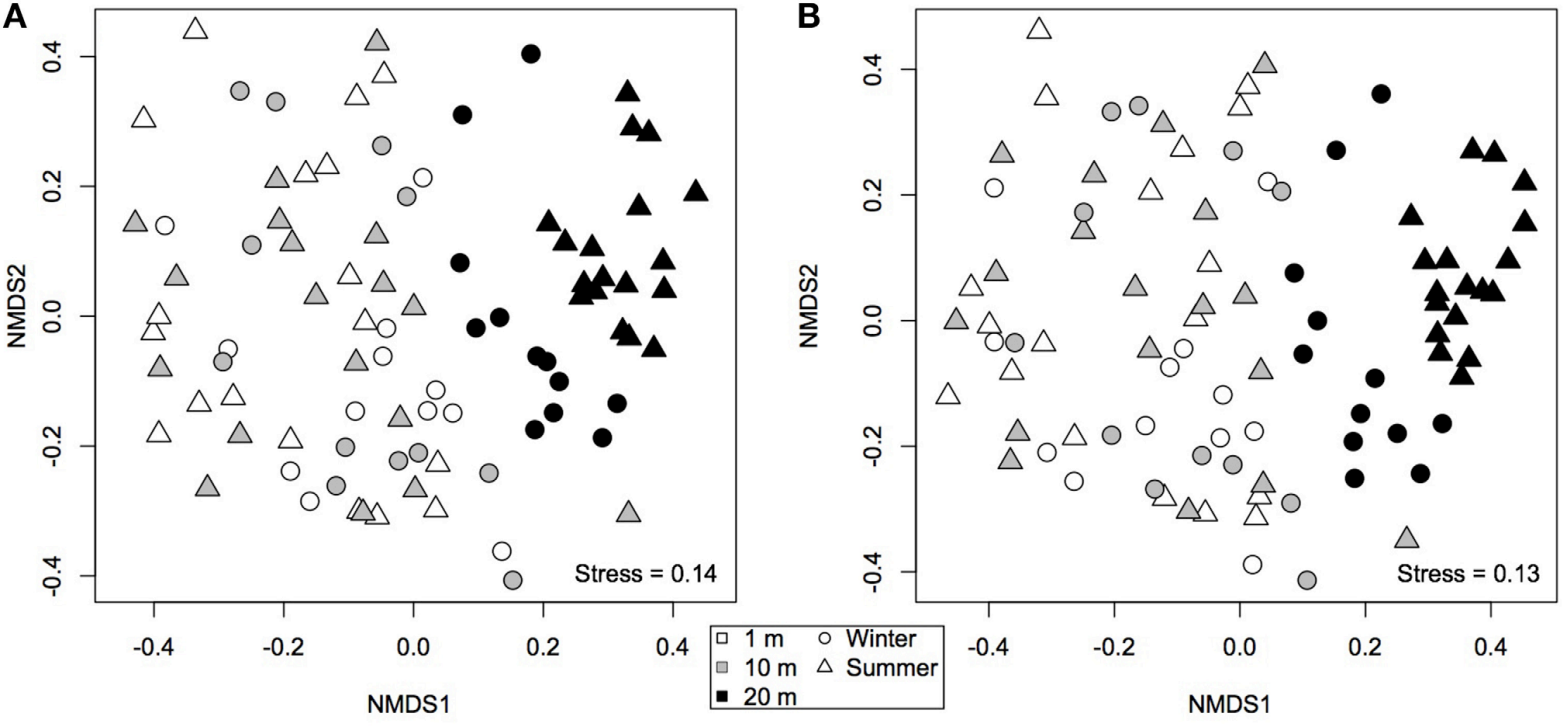
**Non-metric multidimensional scaling ordination based on (A) Bray-Curtis and (B) modified Raup-Crick (Chase et al., 2011) dissimilarities**. Communities that are closer together, using modified Raup-Crick dissimilarities, are more deviant from the null expectation, whereas communities that are farther apart are less deviant from the null expectation.

A non-metric multidimensional scaling plot (nMDS) obtained using Bray-Curtis dissimilarities (Figure 3A) clustered samples by distance from the water line and by season, as confirmed by PERMANOVA analysis (Table 2). Community dispersion also differed between habitats, with dry communities during summer showing the lower β-diversity [BETADISPER: *F*_(5, 84)_ = 4.84, *P* < 0.001]. Similar results were obtained using Jaccard's dissimilarities (not shown). A Mantel test showed that there was a weak, but significant, positive correlation between β-diversity and water content (Mantel *r* = 0.2, *P* < 0.001). Water content and β-diversity were higher in the sediments closest to the pan. Water availability can be linked to productivity, which is thought to increase compositional stochasticity by enhancing ecological drift and weakening niche selection (Zhou et al., 2014). Moreover, productivity seems to strengthen priority effects leading to multiple stable equilibria (Chase, 2010).

**Table 2:** **Non parametric analysis of pairwise similarities calculated using Bray Curtis metric**.

**Source of variance**	**Df**	**SS**	**MS**	**F**	**P**
Time	1	0.26	0.26	0.65	0.89
Season	1	1.26	1.26	3.16	0.0012
Site	1	3.04	3.04	7.61	0.0001
Time × site	1	0.34	0.34	0.85	0.25
Season × site	1	0.88	0.88	2.20	0.64
Residuals	84	33.51	0.40		
Total	89	39.20			

Df, degrees of freedom; F, F-test statistic; MS, mean squares; P, proportion of randomization trials with more extreme values of F; SS, sums of squares.

**Figure 4:**
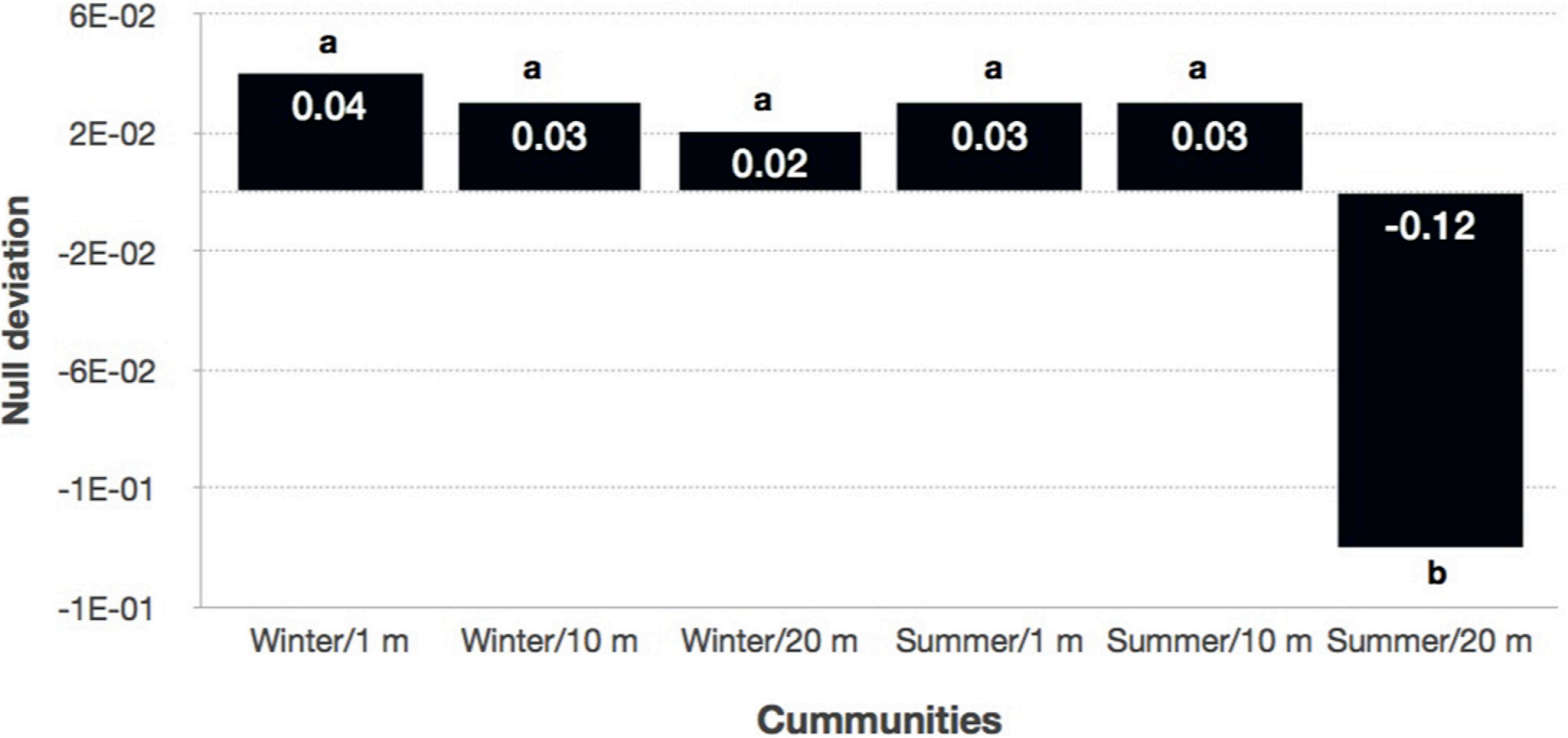
**Plot showing the null deviation (Chase and Myers, 2011) of the bacterial communities grouped by season and distance to the water**. A null deviation close to zero suggests that neutral processes are more important in structuring the community, whereas larger negative null deviations suggest that niche-based processes are more important. Different letters indicate significant differences between samples (*P* < 0.001).

Because these results suggest that both deterministic and stochastic processes are important in explaining bacterial community assembly patterns, but do not provide a mechanistic understanding of the factors that create them, a null model approach was used (Chase et al., 2011). The null approach generated stochastically assembled communities from the regional species pool, providing an indication of the relative contribution of deterministic and stochastic processes. This approach showed that the bacterial communities grouped by season and distance from the water line (Supplementary Table 2). A two-tailed Wilcoxon signed-rank test revealed that the null deviations did not differ from zero in “wet” and “dry/winter” communities (*P* > 0.05), indicating that the observed β-diversity did not differ from random sampling. In contrast, the null deviation was different from zero (null deviation = −0.12; *P* < 0.01) in “dry/summer” communities, reflecting that the observed β-diversity differ from random sampling. A permutational analysis of multivariate dispersion test confirmed that “dry” communities in summer were more similar than expected by chance when compared to the other communities (Figures 3B, 4) [BETADISPER: *F*_(5, 84)_ = 15.41, *P* < 0.001; all pairwise comparisons *P* < 0.001], suggesting a relatively more important role of environmental filtering in shaping “dry/summer” communities relative to “wet” and “dry/winter” communities.

**Figure 5:**
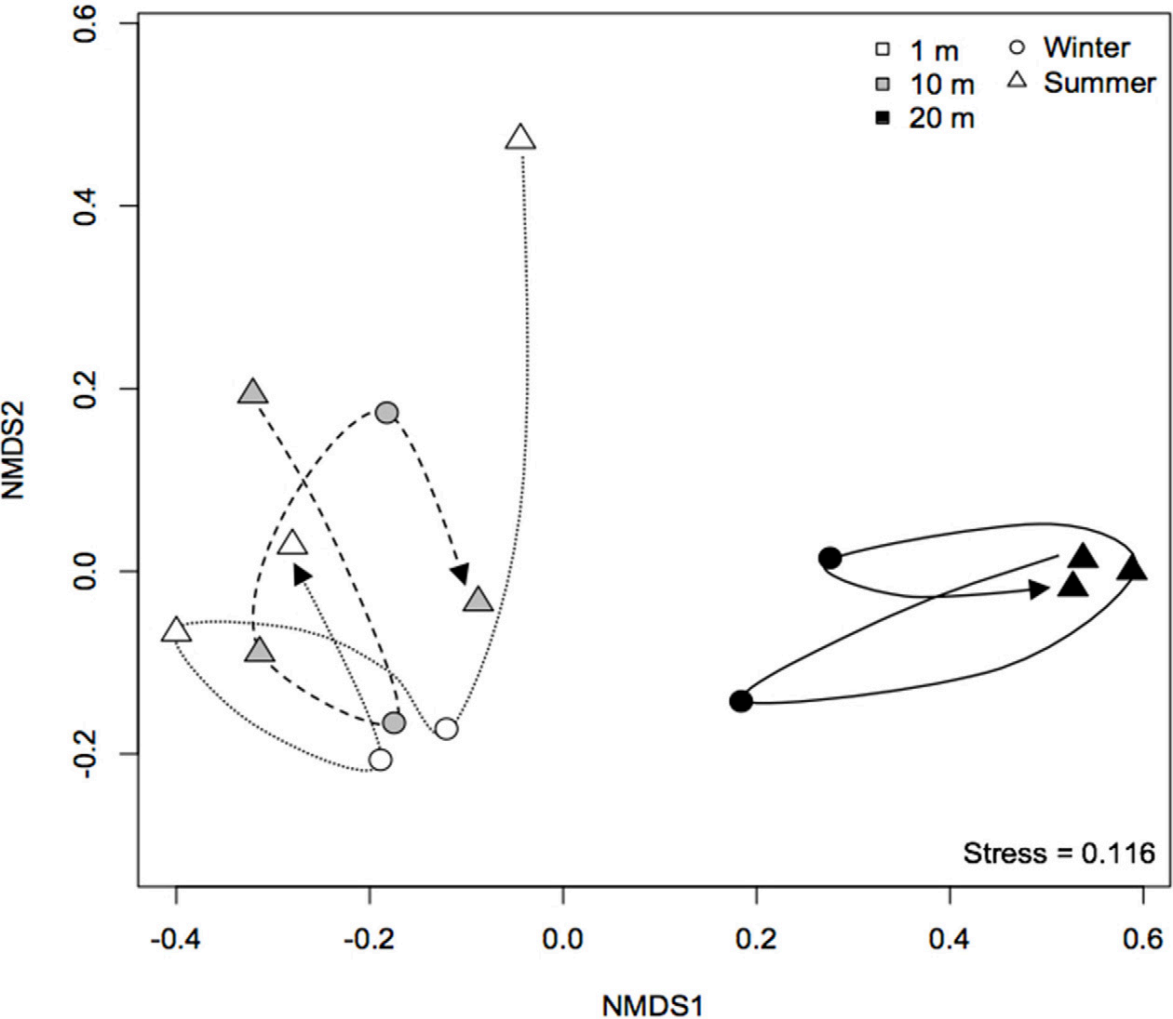
**Non-metric multidimensional scaling plot showing the temporal development of bacterial communities at Yzerfontein salt pan from 2011 to 2014**. The analysis is based on Bray-Curtis dissimilarities of mean relative abundance of OTUs obtained by T-RFLP. The lines connect consecutive sampling occasions.

Furthermore, an nMDS plot showing the temporal development of community composition at Yzerfontein salt pan demonstrated that all “wet” bacterial communities followed a similar trajectory and was clearly different from that of “dry” communities [PERMANOVA: *F*_(1, 13)_ = 6.94, *P* < 0.01], which displayed evidence of cyclical patterns (Figure 5). The cyclical patterns may indicate the resilience of the community to drought (Barnard et al., 2013). Overall, these results are consistent with those from previous studies performed in environments such as ponds (Chase, 2007), deserts (Sheik et al., 2011) and grasslands (Clark et al., 2009), suggesting that desiccation plays an important role in bacterial community assembly. Desiccation, and the consequent reduction in water activity and increase in salinity, imparts considerable osmotic stress on microorganisms and can lead to decreases in cytoplasmic volume, damage to membranes, proteins and nucleic acids and to cellular lysis (Potts, 1999), dramatically affecting bacterial activity (Placella et al., 2012) and community composition (Fierer et al., 2003). Gas exchange from the sediment to atmosphere is altered during the drying process (McKew et al., 2011), increasing diffusion rates and oxygen concentrations in the upper layers, which, in turn, can affect the abundance and composition of anaerobic bacteria (McKew et al., 2011). We did not find N content to vary between samples (Table 1), but nutrients such as phosphorus and nitrogen, parameters that can be related to productivity, have been found to decrease after partial drying of lowland river-floodplain systems (Baldwin and Mitchell, 2000), and are known to shape bacterial β-diversity patterns (Langenheder et al., 2012). Interestingly, bacterial dormancy may be more prevalent in resource limited ecosystems (Jones and Lennon, 2010), a feature that has been shown (Lennon and Jones, 2011) to have the potential to explain two ecological patterns observed here: repeated seasonal succession (Fuhrman et al., 2006), and the resilience of microbial communities (Allison and Martiny, 2008).

We cannot exclude, however, the possibility that these patterns resulted from other factors than desiccation; for instance, abiotic variables that were not recorded and/or biotic interactions (e.g., competition). Alternatively, those patterns could depend on differences in γ-diversity across samples (dry < wet), as β-diversity is known to increase with the richness of the regional species pool. Indeed, this seems to be the case across broad environmental gradients (Kraft et al., 2011), where species assemblages are expected to result from different regional species pools. Nevertheless, given the relatively small spatial scale of our study it seems reasonable to assume that the communities studied here form part of the same metacommunity.

Four distinct mechanisms control community composition within Vellend's conceptual framework: selection, drift, speciation and dispersal. Selection is indubitably deterministic, while ecological drift, speciation and dispersal can be visualized as components of more stochastic processes (Chase and Myers, 2011). The real contribution of dispersal is difficult to evaluate, because the relatively low resolution of our approach makes problematic to conclude that an organism is absent from a specific habitat. However, as the system is open, small and habitats are relatively well connected, dispersal seems not to be a major process influencing community assembly. Laboratory studies have demonstrated that speciation can be relatively easy for bacteria through niche partitioning (e.g., Herron and Doebeli, 2013). However, this might not occur in nature for many microorganisms because, for example, growth can be limited by low nutrient levels. Therefore, the changes we have observed in the bacterial communities in the lateral sediments of Yzertontein salt pan are most likely the result of selection and ecological drift, which is in accordance with recent findings (Stegen et al., 2012, 2013; Zhou et al., 2014). Our results further suggest that the relative influence of deterministic and stochastic processes vary along the desiccation gradient, with deterministic factors being more influential in communities distant from the water line.

### Conflict of interest statement

The authors declare that the research was conducted in the absence of any commercial or financial relationships that could be construed as a potential conflict of interest.
