# Supplementary material for: Contrasting assembly processes in a bacterial metacommunity along a desiccation gradient
Source: Front Microbiol. 2014 Dec 3;5:668. doi: 10.3389/fmicb.2014.00668 (PMC4253974; doi:10.3389/fmicb.2014.00668)

Supplementary Table 1. Sampling dates and climatic data

Sampling dates	Season
16-11-2011	Summer
23-05-2012	Winter
23-11-2012	Summer
06-06-2013	Winter
25-02-2014	Summer

Year	Average temperature min-max (ºC)		Total rainfall (mm)	
	Summer	*Winter*	*Summer*	*Winter*
2011	11-25	7-21	44	188
2012	11-25	6-19	24	263
2013	11-25	6-20	44	332
2014	12-27		52	

	Average wind speed (m/s)		Humidity (%)	
	Summer	*Winter*	*Summer*	*Winter*
2011	3	3	78	88
2012	3	3	78	88
2013	3	3	78	88
2014	3		80	

Supplementary Table 2. Non parametric analysis of pairwise similarities calculated using the modified Raup-Crick dissimilarity metric.

**Source of variance**	**Df**	**SS**	**MS**	**F**	**P**
Time	1	0.26	0.25	1.35	0.29
Season	1	1.16	1.16	6.13	0.0017
Site	1	7.43	7.43	39.19	0.0001
Time x site	1	0.30	0.30	1.63	0.22
Season x site	1	0.09	0.09	0.48	0.65
Residuals	84	15.92	0.19		
Total	89	25.18			

Df, degrees of freedom; F, F-test statistic; MS, mean squares; P, proportion of randomization trials with more extreme values of F; SS, sums of squares.

Supplementary Figure 1. Aerial picture of Yzerfontein salt pan. The picture was obtained with Google Maps.

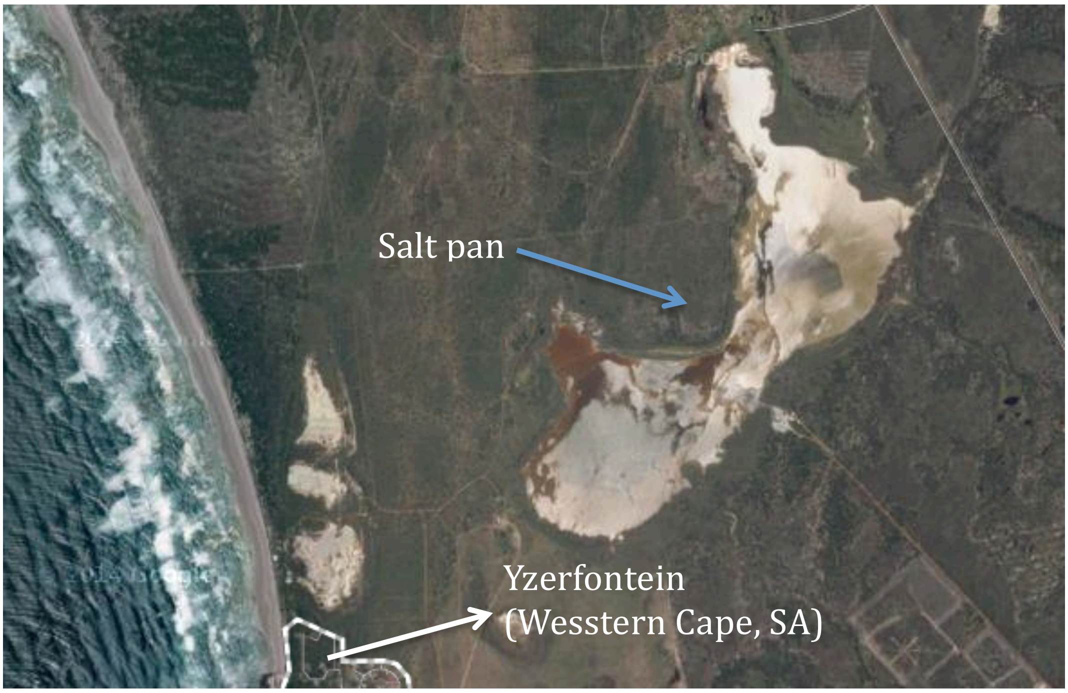

Supplement: Supplementary file 1 [file Presentation1.PDF]
